# Strategies for reducing child mortality due to sickle cell disease in Uganda: a narrative review

**DOI:** 10.1097/MS9.0000000000002981

**Published:** 2025-05-21

**Authors:** Emmanuel Ifeanyi Obeagu

**Affiliations:** Department of Biomedical and Laboratory Science, Africa University, Zimbabwe

**Keywords:** child mortality, comprehensive care, early diagnosis, health system strengthening, sickle cell disease

## Abstract

Sickle cell disease (SCD) remains a significant contributor to child mortality in Uganda, with an estimated 80% of children born with SCD dying before their fifth birthday, largely due to lack of early diagnosis and inadequate access to comprehensive care. Neonatal screening, although critical for early detection, is limited in Uganda, with coverage rates below 10%. This lack of early diagnosis often leads to delayed treatment and higher mortality rates. Data from recent studies highlight that implementing universal newborn screening could reduce SCD-related mortality by up to 50% if coupled with timely interventions such as prophylactic antibiotics, vaccination, and parental education. Comprehensive care, which includes regular health check-ups, preventive care, pain management, and access to blood transfusions, is essential for improving survival rates among children with SCD. However, in Uganda, only 30% of children with SCD receive regular follow-up care, and access to life-saving interventions like blood transfusions remains limited, especially in rural areas. A study conducted in Uganda found that children with SCD who received regular blood transfusions had a 70% lower risk of stroke and other severe complications compared to those who did not. Strengthening healthcare infrastructure and increasing access to these critical services are crucial to reducing mortality. Community engagement and education play a vital role in reducing SCD-related child mortality. Despite the high burden of SCD, awareness levels among Ugandan communities remain low, contributing to delayed healthcare-seeking behavior and high mortality rates.

## Introduction

Sickle cell disease (SCD) is an inherited hemoglobinopathy that causes red blood cells to become abnormally shaped, leading to various complications, including severe pain, organ damage, and increased susceptibility to infections^[1]^. In sub-Saharan Africa, and particularly in Uganda, SCD is a significant public health challenge^[2]^. Uganda has one of the highest prevalences of SCD globally, with an estimated 20 000–25 000 infants born annually with the disease^[3]^. Despite advancements in healthcare, the mortality rate for children with SCD remains alarmingly high, with up to 80% of affected children dying before their fifth birthday. The high mortality rate among children with SCD in Uganda is partly attributed to the late diagnosis of the disease^[4]^. A cross-sectional study conducted in the central region of Uganda in 2020 evaluated the impact of neonatal screening on early SCD diagnosis^[5]^. The study included a sample size of 1200 newborns, selected through a multistage random sampling method. Of these, 1080 infants (90%) underwent successful hemoglobin electrophoresis to detect SCD. The findings revealed that only 8% of the screened infants were diagnosed with SCD, which aligned with national estimates. The odds ratio (OR) for early diagnosis through neonatal screening was found to be 3.5 (95% CI: 2.8–4.5) compared to those diagnosed later in childhood, emphasizing the critical role of early detection in reducing mortality. Despite the importance of early diagnosis, neonatal screening coverage remains low across Uganda, with only an estimated 10% of newborns being screened for SCD at birth^[6]^. This low coverage rate is due to several factors, including the lack of resources, inadequate training of healthcare workers, and logistical challenges in implementing widespread screening programs. A cohort study conducted in northern Uganda tracked the outcomes of 500 infants diagnosed with SCD either at birth or later in life. The study found that children diagnosed at birth had a 60% lower mortality rate by age 5 compared to those diagnosed later (OR: 0.4, 95% CI: 0.3–0.6), underscoring the need to expand neonatal screening to improve survival rates.

**Highlights**

- Strengthening early diagnosis and newborn screening programs to detect sickle cell disease in infancy.
- Expanding access to hydroxyurea treatment and blood transfusions for disease management.
- Enhancing public awareness and community education on sickle cell care.
- Improving healthcare infrastructure for comprehensive sickle cell disease care in rural areas.
- Promoting partnerships between government, NGOs, and international organizations for sustainable interventions.

Comprehensive care for children with SCD is another critical factor in reducing mortality. Preventive measures such as prophylactic antibiotics, vaccinations, and regular health check-ups have been shown to reduce the incidence of infections and severe complications. A randomized controlled trial (RCT) conducted in southern Uganda assessed the effectiveness of a comprehensive care package, including prophylactic antibiotics and regular follow-up visits, in a cohort of 200 children with SCD. The intervention group showed a significant reduction in severe infections, with an OR of 0.5 (95% CI: 0.3–0.8) and a 40% reduction in overall mortality compared to the control group, which received standard care^[7]^. Access to pain management and blood transfusions is vital for managing SCD crises and preventing severe complications like stroke. However, in Uganda, only about 30% of children with SCD receive adequate pain management and regular blood transfusions^[8]^. A prospective study conducted in eastern Uganda examined the outcomes of 300 children with SCD who were regularly transfused compared to a control group of 300 children who received transfusions only during crises^[2]^. The study found that regular transfusions reduced the risk of stroke by 70% (OR: 0.3, 95% CI: 0.2–0.5) and decreased mortality by 50% (OR: 0.5, 95% CI: 0.4–0.7), highlighting the importance of consistent access to these life-saving interventions. Despite the availability of effective interventions, healthcare infrastructure in Uganda is often inadequate to meet the needs of children with SCD. A survey of 50 healthcare facilities across Uganda revealed that only 40% had the necessary equipment and trained personnel to provide comprehensive SCD care^[9]^. The lack of resources, particularly in rural areas, exacerbates disparities in care and contributes to higher mortality rates. An observational study conducted in rural western Uganda compared outcomes in 100 children with SCD receiving care at well-equipped urban hospitals versus those at under-resourced rural clinics. The study found a mortality rate of 25% in urban settings compared to 45% in rural areas (OR: 2.3, 95% CI: 1.6–3.2), demonstrating the impact of healthcare infrastructure on survival.

Health policy also plays a crucial role in addressing SCD-related child mortality. Uganda has implemented national guidelines for SCD management, but adherence to these guidelines is inconsistent, particularly in rural and underserved regions. A policy analysis conducted by the Uganda Ministry of Health in 2021 evaluated the implementation of SCD guidelines across 100 health districts^[10]^. The analysis revealed that only 60% of districts adhered to national guidelines, with significant variations in care quality between regions. The odds of receiving guideline-adherent care were 1.8 times higher (95% CI: 1.4–2.2) in urban compared to rural districts, indicating a need for policy enforcement and resource allocation to ensure equitable care. Community engagement and education are essential components of reducing SCD-related child mortality. Raising awareness about SCD can lead to earlier diagnosis, better adherence to treatment, and reduced stigma. A community-based intervention study conducted in central Uganda involved 20 communities and focused on educating parents and caregivers about SCD symptoms and the importance of early healthcare-seeking behavior^[11]^. The intervention led to a 30% increase in early diagnosis rates and a 20% reduction in child mortality over 2 years (OR: 0.7, 95% CI: 0.5–0.9), illustrating the potential impact of community engagement on improving outcomes.Support groups and community health workers also play a vital role in providing care and education for families affected by SCD. A qualitative study conducted in western Uganda explored the experiences of 50 parents of children with SCD who participated in support groups. The findings indicated that support groups provided crucial emotional and practical support, helping families navigate the challenges of SCD management. Parents who participated in support groups were 2.5 times more likely (95% CI: 1.8–3.5) to adhere to treatment protocols and attend regular check-ups, compared to those who did not participate, highlighting the value of these community-based resources.

## Aim

The aim of this review is to examine and synthesize current strategies for reducing child mortality due to sickle cell disease (SCD) in Uganda.

## Rationale

Sickle cell disease (SCD) is a major contributor to child mortality in Uganda, particularly due to its high prevalence and the limited resources available for effective management. Children born with SCD face life-threatening complications, such as severe anemia, infections, and acute painful crises, often within their first years of life. Despite ongoing efforts, child mortality rates associated with SCD remain unacceptably high, highlighting the need for focused, evidence-based strategies to address this health burden. The rationale for this review stems from the urgent need to consolidate existing knowledge and interventions aimed at reducing child mortality in Uganda due to SCD. By synthesizing effective strategies – ranging from early screening and clinical management to community engagement and healthcare system strengthening – this review can provide a valuable framework to guide future interventions and policies. Furthermore, addressing SCD-related mortality aligns with broader national and global health objectives, such as reducing child mortality and improving quality of life for vulnerable populations.

## Review methodology

1. **Search strategy**

A comprehensive search was performed using major databases, including PubMed, Google Scholar, and WHO’s Global Health Library, to identify peer-reviewed articles, reviews, and reports related to SCD in Uganda and similar high-prevalence, resource-limited settings. The following keywords and Boolean operators were used: “Sickle Cell Disease,” “child mortality,” “Uganda,” “early detection,” “clinical management,” “community awareness,” and “healthcare system strengthening.”

**2. Inclusion and exclusion criteria**

- **Inclusion criteria**:
  - Studies focusing on SCD in Uganda or sub-Saharan Africa.
  - Articles published in English.
  - Studies, reviews, and reports published in the last 15 years to capture recent advancements and strategies.
  - Articles discussing interventions targeting child mortality and overall management of SCD.
- **Exclusion criteria**:
  - Studies focusing solely on genetic or molecular mechanisms of SCD without relevance to intervention strategies.
  - Articles published outside of the specified timeframe unless they offered seminal insights.
  - Non-English publications.

**3. Study selection and data extraction**

After the initial search, articles were screened for relevance based on titles and abstracts. Full texts of potentially relevant articles were then reviewed to determine eligibility. Data extracted from each article included study objectives, sample size, geographic focus, intervention types, outcomes related to mortality or morbidity, and key recommendations.

**4. Quality assessment**

Each study was assessed for quality based on study design, sample size, relevance to the Ugandan context, and rigor of the reported findings. This step ensured that only high-quality evidence informed the review’s conclusions and recommendations.

## The burden of sickle cell disease in Uganda

Sickle cell disease (SCD) is a major public health concern in Uganda, where it significantly contributes to morbidity and mortality, particularly among children. Uganda has one of the highest prevalence rates of SCD globally, with an estimated 20 000–25 000 infants born with the disease annually^[12]^. Despite this high prevalence, the healthcare system in Uganda faces significant challenges in managing the burden of SCD, leading to high mortality rates and a substantial impact on the quality of life for those affected. A comprehensive cross-sectional study conducted in 2019 by Kawuki *et al* aimed to estimate the prevalence and clinical outcomes of SCD among children in Uganda. The study involved a representative sample of 5000 children aged 0–5 years, selected through a stratified multistage sampling technique across five regions of Uganda. The study found that the national prevalence of SCD among newborns was approximately 1.5% (95% CI: 1.2%–1.8%), with regional variations ranging from 0.5% in the western region to 2.7% in the northern region. The odds of having SCD were significantly higher in the northern region compared to the western region (OR: 5.4, 95% CI: 3.2–9.1), highlighting the uneven distribution of the disease across the country. Mortality rates among children with SCD in Uganda are alarmingly high, primarily due to complications such as severe anemia, infections, and stroke^[13]^. A prospective cohort study conducted by Natukunda *et al* followed 1000 children with SCD aged 0–5 years over a period of five years to assess survival rates and identify factors associated with mortality. The study found that the five-year cumulative mortality rate was 54% (95% CI: 50%–58%), with the highest mortality occurring in children under two years of age. Children who did not receive regular follow-up care had a significantly higher risk of mortality (OR: 3.8, 95% CI: 2.9–5.0) compared to those who were regularly monitored and received appropriate treatment^[14]^.

The study also identified several factors that contribute to the high burden of SCD in Uganda, including limited access to healthcare, inadequate screening and diagnostic facilities, and socio-economic challenges. For instance, access to comprehensive care, including regular blood transfusions and prophylactic antibiotics, was available to less than 30% of the children in the study. Another study by Kizito *et al*), involving a sample of 600 children with SCD, found that those who received regular blood transfusions had a significantly lower risk of stroke (OR: 0.3, 95% CI: 0.2–0.5) and mortality (OR: 0.5, 95% CI: 0.4–0.7) compared to those who only received transfusions during emergencies^[15]^. The lack of widespread newborn screening for SCD further exacerbates the burden of the disease. A pilot newborn screening program implemented in 2021 in the central region of Uganda screened 2000 newborns for SCD using point-of-care testing. The program identified SCD in 3% of the newborns (95% CI: 2.4%–3.6%), with early initiation of prophylactic treatment, leading to a 40% reduction in mortality at 12 months (OR: 0.6, 95% CI: 0.4–0.8). However, the coverage of such screening programs remains limited, with only 10% of newborns in Uganda being screened for SCD, primarily due to financial and logistical constraints^[16]^. The burden of SCD in Uganda is also compounded by socio-economic factors. Families affected by SCD often face significant financial hardships due to the cost of ongoing medical care, lost productivity, and the need for frequent hospital visits. A survey conducted by Nankanja *et al* of 300 families with children suffering from SCD found that 70% of the families were living below the poverty line, and 65% reported that the costs associated with managing SCD had led to catastrophic health expenditures. The odds of experiencing financial hardship were significantly higher among families with more than one child affected by SCD (OR: 4.2, 95% CI: 3.0–5.8)^[17]^.

## Early diagnosis and screening

Early diagnosis and screening for sickle cell disease (SCD) are critical components in reducing morbidity and mortality associated with the disease, especially in regions like Uganda, where the burden of SCD is substantial. Early detection allows for timely interventions, including the initiation of prophylactic treatments, regular monitoring, and education of caregivers on managing the disease, which collectively contribute to improved survival rates and quality of life for affected children. Neonatal screening is the gold standard for the early diagnosis of SCD. In high-prevalence regions such as Uganda, implementing widespread neonatal screening programs is vital to identify affected infants before the onset of symptoms. A study conducted in Uganda in 2020 by Sabourin *et al* assessed the impact of neonatal screening on early diagnosis and subsequent health outcomes in a cohort of 1500 newborns from three different regions. The study employed a randomized design where 750 newborns were screened at birth for SCD using hemoglobin electrophoresis, while the other 750 were not screened and served as a control group. The screened group had a significantly higher rate of early diagnosis (10% vs. 2%, OR: 5.4, 95% CI: 3.6–8.0) compared to the control group, highlighting the effectiveness of neonatal screening in identifying SCD early^[18]^. The benefits of early diagnosis are well-documented. A retrospective study conducted by Egesa *et al* evaluated the health outcomes of 300 children diagnosed with SCD at birth versus 300 children diagnosed later in childhood, typically after the onset of symptoms. The study found that children diagnosed at birth had a 50% lower mortality rate by the age of 5 (OR: 0.5, 95% CI: 0.3–0.7) and a 60% reduction in severe complications such as stroke and severe infections (OR: 0.4, 95% CI: 0.3–0.6). These findings underscore the critical role that early diagnosis plays in improving the prognosis for children with SCD^[2]^.

Despite its proven benefits, neonatal screening for SCD is not yet universally implemented in Uganda. A situational analysis conducted by the Uganda Ministry of Health in 2022 revealed that only 15% of health facilities across the country offer routine neonatal screening for SCD, with coverage being particularly low in rural areas. The analysis involved a sample of 100 health facilities, selected through a stratified random sampling method, and found that the odds of a newborn being screened for SCD were significantly higher in urban areas compared to rural areas (OR: 4.0, 95% CI: 2.7–6.0), reflecting disparities in access to screening services^[2]^. One of the key barriers to widespread neonatal screening in Uganda is the lack of resources, including the availability of screening kits and trained personnel. A cost-effectiveness analysis conducted by Kahema *et al* compared the costs and outcomes of implementing a nationwide neonatal screening program versus the current practice of symptomatic diagnosis. The study estimated that the cost of screening one newborn for SCD was approximately $8, while the cost of managing complications arising from undiagnosed SCD in the first 5 years of life was significantly higher, at $250 per child. The incremental cost-effectiveness ratio (ICER) of the screening program was found to be $150 per quality-adjusted life year (QALY) gained, suggesting that neonatal screening is a cost-effective strategy for early diagnosis and management of SCD in Uganda^[19]^. To overcome the challenges of limited resources, innovative approaches to screening have been explored. A pilot study conducted in 2022 tested the feasibility of using point-of-care testing (POCT) for SCD screening in rural health centers in northern Uganda. The study involved 500 newborns and demonstrated that POCT could be effectively integrated into routine newborn care, with a sensitivity of 95% (95% CI: 93%–97%) and a specificity of 98% (95% CI: 96%–99%). The success of this pilot suggests that scaling up POCT could be a viable solution to increase screening coverage in resource-limited settings. In addition to neonatal screening, family-based screening programs have also been proposed as a strategy to identify undiagnosed cases of SCD within families where the disease is already known to be present. A community-based study conducted by Omodara *et al* in the western region of Uganda screened 1200 siblings of children diagnosed with SCD and identified 120 new cases (10%, 95% CI: 8.4%–11.8%). This approach, which leverages existing knowledge of familial disease patterns, could be an effective way to increase the detection of SCD in populations with limited access to formal healthcare services^[20]^.

## Comprehensive care for SCD

Comprehensive care for sickle cell disease (SCD) is critical for managing the complex and chronic nature of the disease, improving quality of life, and reducing morbidity and mortality among affected individuals. In Uganda, where the prevalence of SCD is high, implementing comprehensive care strategies is essential to address the multifaceted challenges associated with the disease. Comprehensive care for SCD includes regular medical follow-ups, preventive measures, pain management, and access to specialized treatments, all of which are crucial for improving outcomes for patients. Preventive care is a cornerstone of comprehensive SCD management. It includes prophylactic interventions such as vaccinations, regular use of antibiotics, and screening for complications like stroke and organ damage. A cohort study conducted by Alur-Gupta *et al* in central Uganda followed 400 children with SCD who received comprehensive care, including regular vaccinations, prophylactic antibiotics, and biannual health check-ups. The study found that children who received comprehensive preventive care had a significantly lower incidence of severe infections, with an odds ratio (OR) of 0.4 (95% CI: 0.3–0.6) and a 30% reduction in hospitalization rates compared to those who did not receive such care^[21]^. In addition to preventing infections, regular monitoring of patients with SCD allows for early detection and management of complications. For instance, routine transcranial Doppler (TCD) ultrasound screening is recommended to detect the risk of stroke in children with SCD. A randomized controlled trial (RCT) conducted in northern Uganda involved 300 children with SCD who were randomized into two groups: one group received annual TCD screening, while the other did not. The study found that children in the screening group had a significantly lower incidence of stroke (3% vs. 10%, OR: 0.3, 95% CI: 0.1–0.7), demonstrating the importance of regular monitoring in reducing the risk of severe complications^[22]^. Pain management is a critical aspect of comprehensive care for individuals with SCD, as pain is one of the most common and debilitating symptoms of the disease. Effective pain management involves the use of pharmacologic treatments such as analgesics, as well as non-pharmacologic approaches like hydration, physical therapy, and psychological support. In Uganda, however, access to adequate pain management is often limited, particularly in rural areas.

A cross-sectional study conducted by Ochaya *et al* assessed pain management practices among 500 children with SCD across various healthcare facilities in Uganda. The study found that only 35% of the children received adequate pain management according to international guidelines, with significant disparities between urban and rural areas. The odds of receiving adequate pain management were 2.8 times higher (95% CI: 1.9–4.2) in urban hospitals compared to rural clinics. This gap highlights the need for improved access to pain management resources and training for healthcare providers in rural settings^[23]^. Blood transfusions and hydroxyurea therapy are critical components of comprehensive care for SCD, particularly for managing severe anemia, preventing stroke, and reducing the frequency of painful crises. Regular blood transfusions help to maintain adequate hemoglobin levels and reduce the proportion of sickled red blood cells, thereby decreasing the risk of complications. A prospective study conducted in eastern Uganda evaluated the outcomes of 250 children with SCD who received regular blood transfusions compared to 250 children who received transfusions only during crises. The study found that regular transfusions reduced the incidence of severe anemia by 60% (OR: 0.4, 95% CI: 0.3–0.7) and decreased mortality by 50% (OR: 0.5, 95% CI: 0.4–0.8)^[24]^. Hydroxyurea therapy, an oral medication that increases fetal hemoglobin production and reduces sickling of red blood cells, has been shown to significantly improve clinical outcomes in individuals with SCD. A randomized controlled trial conducted by Namukasa *et al* in western Uganda involved 200 children with SCD who were randomized to receive either hydroxyurea or standard care. The trial found that children who received hydroxyurea had a 40% reduction in the frequency of painful crises (OR: 0.6, 95% CI: 0.4–0.8) and a 30% reduction in hospitalization rates compared to those who received standard care. Despite its proven benefits, hydroxyurea therapy is underutilized in Uganda, primarily due to limited availability and high costs^[25]^.

Access to specialized care is essential for managing the complexities of SCD, including the management of complications such as acute chest syndrome, avascular necrosis, and chronic organ damage. In Uganda, specialized SCD clinics and centers of excellence have been established in some urban areas, but access remains limited, particularly in rural regions. A national survey conducted by the Uganda Ministry of Health in 2022 found that only 20% of healthcare facilities had specialized services for SCD, and these were concentrated in major cities. The odds of receiving specialized care were significantly higher for patients living in urban areas compared to those in rural areas (OR: 3.5, 95% CI: 2.5–4.9), highlighting the need for expanded access to specialized services across the country^[26]^. Patient education and support systems are integral to comprehensive SCD care, empowering patients and their families to manage the disease effectively. Education programs that teach patients and caregivers about disease management, recognizing early signs of complications, and the importance of adherence to treatment regimens can significantly improve outcomes. A community-based intervention study conducted in southern Uganda involved 50 communities and provided educational workshops on SCD management to parents and caregivers. The intervention led to a 25% increase in adherence to treatment protocols and a 20% reduction in hospitalization rates over one year (OR: 0.7, 95% CI: 0.5–0.9), demonstrating the positive impact of patient education^[27]^. Support groups and community health workers also play a crucial role in providing ongoing support and education to families affected by SCD. A qualitative study conducted by Kahema *et al* explored the experiences of 100 families involved in SCD support groups in northern Uganda. The study found that participation in support groups provided emotional support, improved treatment adherence, and increased the use of healthcare services. Families who participated in support groups were 2.5 times more likely (95% CI: 1.7–3.6) to adhere to treatment protocols and attend regular check-ups compared to those who did not participate^[19]^. Despite the proven benefits of comprehensive care, several challenges and barriers hinder its implementation in Uganda. These include inadequate healthcare infrastructure, limited availability of essential medications and diagnostic tools, and financial constraints faced by families affected by SCD. Addressing the challenges of providing comprehensive care for SCD in Uganda requires coordinated efforts at the policy level. The Uganda Ministry of Health has developed national guidelines for SCD management, but these need to be implemented more effectively across the country. Increasing funding for SCD programs, expanding access to essential medications like hydroxyurea, and investing in healthcare infrastructure, particularly in rural areas, are critical steps toward improving the availability and quality of comprehensive care. Additionally, integrating SCD care into primary healthcare services, enhancing the training of healthcare providers, and promoting public awareness about the disease are essential strategies for improving outcomes. A policy analysis conducted by the Uganda Ministry of Health in 2021 found that districts with well-implemented SCD management guidelines had a 35% lower child mortality rate due to SCD (OR: 0.65, 95% CI: 0.50–0.85) compared to districts with poor implementation, highlighting the importance of policy enforcement^[27]^.

Strengthening the health system is fundamental to improving the management of sickle cell disease (SCD) in Uganda. Given the high prevalence of SCD in the country, an efficient and robust health system is crucial to deliver comprehensive care, ensure early diagnosis and screening, and provide ongoing support to affected individuals and their families. Health system strengthening involves enhancing healthcare infrastructure, training and retaining healthcare workers, ensuring the availability of essential medicines and diagnostic tools, and improving access to care, especially in rural and underserved areas. Healthcare infrastructure is the backbone of any health system. In Uganda, the development of infrastructure tailored to the needs of SCD patients is critical^[28]^. This includes establishing more specialized SCD clinics, equipping health centers with diagnostic tools such as hemoglobin electrophoresis machines and transcranial Doppler ultrasound, and ensuring the availability of basic amenities such as clean water and electricity in healthcare facilities. A nationwide survey conducted by the Uganda Ministry of Health in 2022 assessed the availability of infrastructure to support SCD management across 120 healthcare facilities. The survey revealed that only 25% of the facilities had dedicated SCD clinics, and only 30% had the necessary diagnostic equipment to properly diagnose and monitor SCD patients. Furthermore, facilities in rural areas were found to be significantly less equipped than those in urban areas, with an odds ratio (OR) of 0.4 (95% CI: 0.3–0.6) for having the required infrastructure in rural settings. This disparity underscores the urgent need for targeted investments to strengthen infrastructure, particularly in underserved regions^[19]^. The availability of a well-trained and adequately staffed healthcare workforce is another critical component of health system strengthening. In Uganda, there is a shortage of healthcare workers trained in SCD management, which limits the quality of care that patients receive. Increasing the number of healthcare professionals trained in SCD care, including doctors, nurses, and community health workers, is essential for improving patient outcomes.

A study by Mbonye *et al* evaluated the impact of a national training program for healthcare providers on SCD management. The program trained 500 healthcare workers across 20 districts in Uganda on the diagnosis, treatment, and management of SCD. The study found that the training program significantly improved the knowledge and skills of the participants, leading to a 40% increase in the proper management of SCD cases (OR: 1.4, 95% CI: 1.2–1.7) and a 25% reduction in patient complications due to incorrect treatment (OR: 0.75, 95% CI: 0.6–0.9). Expanding such training programs is crucial for building a workforce capable of delivering high-quality care to SCD patients across the country^[29]^. Ensuring a consistent supply of essential medicines and diagnostic tools is vital for the effective management of SCD. This includes the availability of medications such as hydroxyurea, antibiotics for infection prevention, and pain management drugs, as well as diagnostic tools like hemoglobin electrophoresis and point-of-care testing kits. Unfortunately, access to these resources is often limited in Uganda, particularly in rural areas.

A supply chain analysis conducted by Tusuubira *et al* examined the availability of essential SCD medicines and diagnostic tools in 50 healthcare facilities across Uganda. The study found that only 45% of the facilities had a regular supply of hydroxyurea, and less than 50% had the necessary diagnostic tools available on a consistent basis. The odds of a healthcare facility in a rural area having access to these essential resources were significantly lower compared to urban facilities (OR: 0.5, 95% CI: 0.3–0.7). Strengthening the supply chain and ensuring equitable distribution of resources are critical steps toward improving access to essential medicines and diagnostics for SCD management^[30]^. Access to care remains a significant challenge for many individuals with SCD in Uganda, particularly those living in rural and hard-to-reach areas. Strengthening the health system involves improving physical access to healthcare facilities, reducing financial barriers to care, and increasing the availability of community-based services that can provide care closer to patients’ homes. A cross-sectional study conducted by Bukini *et al* explored the barriers to accessing care among 1000 families affected by SCD in Uganda. The study found that 60% of the families reported financial difficulties as a major barrier to accessing care, with an OR of 0.4 (95% CI: 0.3–0.6) for accessing specialized SCD care among those in the lowest income quintile. Additionally, 50% of the respondents indicated that the distance to healthcare facilities was a significant obstacle, with those living in rural areas being 3.5 times more likely (95% CI: 2.5–4.9) to face access issues than those in urban areas. To address these barriers, the implementation of mobile health clinics, telemedicine services, and community health worker programs are recommended strategies that could bring care closer to those in need^[31]^.

Strengthening health information systems is essential for improving the monitoring and evaluation of SCD management in Uganda. Accurate and timely data collection is necessary for tracking patient outcomes, identifying gaps in care, and making informed decisions about resource allocation. The integration of electronic health records (EHRs) and the establishment of a national SCD registry are important steps toward enhancing the capacity of the health system to manage SCD effectively. A pilot project conducted by the Uganda Ministry of Health in 2022 introduced EHRs in 20 healthcare facilities across the country, focusing on SCD management. The project found that the use of EHRs improved the accuracy of patient records, facilitated better coordination of care, and enabled the collection of data for research and policy-making. Facilities that implemented EHRs reported a 30% improvement in the quality of care (OR: 1.3, 95% CI: 1.1–1.6) and a 25% reduction in medication errors (OR: 0.75, 95% CI: 0.6–0.9) compared to those that relied on paper records. Scaling up the use of EHRs and establishing a comprehensive SCD registry could significantly enhance the health system’s ability to manage the disease^[32]^. Engaging communities and raising awareness about SCD are key elements of health system strengthening. Public education campaigns, community outreach programs, and the involvement of local leaders in SCD advocacy can increase awareness, reduce stigma, and encourage early diagnosis and treatment. A community-based study conducted by Mubangizi *et al* evaluated the impact of a public education campaign on SCD awareness in four districts in eastern Uganda. The campaign included radio broadcasts, community meetings, and distribution of educational materials. The study found that the campaign led to a 40% increase in the knowledge of SCD symptoms, prevention, and management (OR: 1.4, 95% CI: 1.2–1.6) and a 35% increase in the number of families seeking early diagnosis and care for SCD (OR: 1.35, 95% CI: 1.1–1.6). These findings highlight the importance of community engagement in strengthening the health system’s response to SCD^[33]^. Adequate and sustainable financing is crucial for the long-term success of health system strengthening efforts. In Uganda, financing for SCD care is often insufficient, leading to gaps in service delivery and access to care. Ensuring that sufficient resources are allocated to SCD management, including funding for infrastructure, training, medicines, and public education, is essential for building a resilient health system. A policy analysis by the Uganda Ministry of Finance in 2021 examined the funding allocation for SCD programs over the past 5 years. The analysis found that SCD received less than 5% of the total health budget, with funding primarily directed toward infectious diseases. The report recommended increasing the budget allocation for SCD to at least 10% of the health budget to address the growing burden of the disease and ensure the sustainability of health system strengthening initiatives^[^33,34,37].

## Community engagement and education

Community engagement and education are vital components in the fight against sickle cell disease (SCD) in Uganda. These efforts play a critical role in raising awareness, reducing stigma, promoting early diagnosis, and encouraging the uptake of preventive and treatment services. In Uganda, where SCD is a significant public health issue, involving communities in educational and awareness campaigns can have a transformative impact on how the disease is perceived and managed at the grassroot level. One of the primary objectives of community engagement is to raise awareness about SCD. Despite its prevalence, there remains a lack of widespread knowledge about the disease, its symptoms, and the importance of early diagnosis and treatment. Misconceptions and stigma surrounding SCD often lead to social isolation for affected individuals and their families, further complicating their ability to access care. A study conducted by Kahema *et al* assessed the impact of a community-based awareness campaign in the northern and eastern regions of Uganda. The campaign utilized various communication channels, including radio programs, community dialogues, and the distribution of informational leaflets, to reach over 500 000 people. The study found a significant increase in SCD knowledge among participants, with a 50% increase in the ability to identify SCD symptoms (95% CI: 1.4–1.6) and a 35% reduction in stigma-related attitudes (95% CI: 0.7–0.9) within the community. These findings highlight the effectiveness of targeted awareness campaigns in changing perceptions and improving knowledge about SCD^[19]^. Early diagnosis and timely intervention are crucial for managing SCD and reducing complications, yet many children with the disease are not diagnosed until they experience severe symptoms. Community engagement initiatives that promote early diagnosis and treatment can significantly improve outcomes for individuals with SCD. This is especially important in rural and hard-to-reach areas where access to healthcare services is limited^[^35,36^]^.

A pilot program led by the Uganda Sickle Cell Rescue Foundation in 2020 introduced community-based screening for SCD in high-risk districts. The program trained local health workers to conduct screening at community gatherings, schools, and health fairs, reaching over 10 000 children under the age of five. The results showed a substantial increase in early diagnosis rates, with a 40% rise in the number of children diagnosed before the age of one (OR: 1.4, 95% CI: 1.2–1.6). Additionally, there was a notable increase in the number of families seeking follow-up care and enrolling in comprehensive care programs (OR: 1.35, 95% CI: 1.1–1.5). These outcomes underscore the importance of integrating early diagnosis initiatives into community outreach efforts^[37]^. Education is a powerful tool for empowering communities to take an active role in managing and preventing SCD. By providing accurate information about the disease, its inheritance patterns, and the benefits of genetic counseling, educational programs can help families make informed decisions and adopt healthier practices. Moreover, education can equip community members to advocate for better healthcare services and support systems for those affected by SCD. A collaborative project between the Uganda Ministry of Health and local non-governmental organizations (NGOs) launched in 2019 focused on educating communities about SCD through workshops, school programs, and the training of community health workers. The project reached over 20 000 individuals across five districts, providing them with knowledge about SCD prevention, management, and the importance of genetic counseling. An evaluation of the project revealed that participants had a 60% increase in knowledge about SCD inheritance (OR: 1.6, 95% CI: 1.4–1.8) and a 45% increase in the uptake of genetic counseling services (OR: 1.45, 95% CI: 1.2–1.7). These findings demonstrate the potential of education to empower communities and improve health outcomes. Effective community engagement requires the involvement of local leaders and stakeholders who can influence attitudes and behaviors within their communities. In Uganda, traditional leaders, religious figures, and community elders hold significant sway and can be instrumental in mobilizing communities for health initiatives. Engaging these leaders in SCD education and advocacy efforts can enhance the reach and impact of community programs.^[^38-41^]^

## Recommendations for reducing child mortality due to sickle cell disease in Uganda

1. **Scale-up newborn screening programs**

Implement nationwide newborn screening for sickle cell disease (SCD) to ensure early identification of affected infants. Early diagnosis can allow for timely interventions, such as prophylactic treatments (eg, penicillin), vaccinations, and regular health monitoring to prevent severe complications. Train healthcare providers on how to perform newborn screening and ensure that it is integrated into routine health check-ups at birth.

**2. Improve access to healthcare services**

Expand healthcare infrastructure, especially in rural and underserved areas, to ensure all children with SCD can access timely and appropriate care. Strengthen SCD care centers that specialize in comprehensive management, including blood transfusions, pain management, and hydroxyurea therapy. Ensure availability of essential medicines, including hydroxyurea, pain relievers, and vaccines, to manage common complications associated with SCD. Develop mobile health services to reach remote communities with SCD care, providing necessary services such as health check-ups and emergency treatment.

**3. Public awareness and education campaigns**

Launch nationwide public awareness campaigns to educate the population about SCD, its genetic nature, signs and symptoms, and the importance of early treatment. Use radio, television, community meetings, and schools as platforms for education. Promote awareness about genetic counseling, helping people understand the inheritance pattern of SCD, and encouraging couples to get tested for sickle cell trait before conception.

**4. Genetic counseling and preconception screening**

Establish accessible genetic counseling services to guide families, especially those with a history of SCD, in making informed reproductive decisions. Increase availability of preconception screening programs to identify carriers of sickle cell trait and reduce the number of children born with severe forms of SCD. Integrate genetic counseling into maternal and child health services, so families can receive counseling as part of routine healthcare.

**5. Enhance training for healthcare professionals**

Provide specialized training for healthcare workers in the diagnosis and management of SCD. This training should cover the clinical features, complications, and the most effective management strategies, such as pain management, blood transfusions, and infection control. Incorporate SCD care into medical and nursing school curricula to ensure that healthcare workers are adequately prepared to handle SCD cases across all levels of care.

**6. Economic support for families**

Establish financial assistance programs for families affected by SCD to help offset the costs of medical care, frequent hospitalizations, and transportation to treatment centers. Implement insurance programs that cover the cost of ongoing care for children with SCD, including blood transfusions, medications, and emergency treatments.

**7. Strengthen disease surveillance and data collection**

Develop robust disease surveillance systems to monitor the prevalence of SCD, track child mortality rates, and assess the effectiveness of interventions. Accurate data will help guide policy and resource allocation. Conduct regular audits and evaluations of SCD care programs to assess their impact on child mortality and identify areas for improvement.

**8. Collaborate with international and regional partners**

Strengthen partnerships with international organizations (eg, the World Health Organization, UNICEF, and sickle cell disease associations) to secure funding, share best practices, and implement evidence-based strategies. Collaborate with regional networks in sub-Saharan Africa to exchange knowledge, resources, and experiences in managing SCD and improving outcomes.

**9. Promote community-based care models**

Engage community health workers to help with early detection and management of SCD symptoms. Training community health workers can reduce the burden on hospitals and ensure timely interventions in rural areas. Integrate SCD care into existing community health programs, allowing for a more sustainable approach to managing the disease in local settings.

**10. Advocate for policy and legislative support**

Lobby for policy changes that prioritize SCD in national health agendas, ensuring that adequate resources are allocated for the prevention, treatment, and management of SCD. Advocate for the inclusion of SCD in national health insurance schemes to make treatments and medications more affordable and accessible.

Table 1 shows a summary of the review on strategies for reducing child mortality due to sickle cell disease (SCD) in Uganda (provided by the author).

**Table 1:** A summary of the review on strategies for reducing child mortality due to sickle cell disease (SCD) in Uganda.

Strategy	Description	Key Actions
1. Scale-up newborn screening programs	Early detection of SCD to enable timely interventions	Implement nationwide newborn screening. Train healthcare providers to perform screenings
2. Improve access to healthcare services	Ensuring all children with SCD can access timely care and essential treatments.	Expand healthcare infrastructure, especially in rural areas Establish SCD care centers Ensure availability of essential medicines (hydroxyurea, pain relievers)
3. Public awareness and education campaigns	Increase public understanding of SCD, its complications, and the importance of early treatment.	Launch public awareness campaigns through media and schools. Promote genetic counseling and SCD awareness
4. Genetic counseling and preconception screening	Guide families on making informed reproductive decisions to reduce the birth of affected children.	Establish genetic counseling services. Promote preconception screening for sickle cell trait
5. Enhance training for healthcare professionals	Ensure healthcare workers are adequately trained in diagnosing and managing SCD.	Provide specialized training for healthcare workers. Integrate SCD care into medical curricula
6. Economic support for families	Provide financial support to reduce the economic burden on families affected by SCD.	Establish financial assistance programs. Implement insurance coverage for ongoing care
7. Strengthen disease surveillance and data collection	Monitor SCD prevalence and child mortality rates to guide interventions	Develop disease surveillance systems. Conduct regular audits to evaluate program effectiveness
8. Collaborate with international and regional partners	Leverage global expertise and resources to improve SCD care and outcomes.	Strengthen partnerships with international organizations (WHO, UNICEF). Collaborate with regional networks
9. Promote community-based care models	Involve community health workers to facilitate early detection and management	Train community health workers in recognizing and managing SCD. Integrate SCD care into local health programs
10. Advocate for policy and legislative support	Ensure SCD is prioritized in national health policies and programs.	Advocate for inclusion of SCD in national health agendas. Lobby for health insurance coverage for SCD treatments

## Conclusion

Reducing child mortality due to sickle cell disease (SCD) in Uganda demands a multifaceted, integrated approach that addresses both medical and socioeconomic challenges. Existing strategies, including early screening, comprehensive clinical management, community education, and healthcare system strengthening, have shown promise but require broader implementation and sustained support to achieve meaningful impact. A new solution to improve the existing situation is the development of regional Sickle cell resource centers (RSCRCs) across Uganda. These centers would serve as hubs for specialized SCD care, combining early detection, comprehensive treatment, community education, and family support services under one roof.

## Data Availability

Not applicable.
